# A Propidium Monoazide (PMAxx)-Droplet Digital PCR (ddPCR) for the Detection of Viable *Burkholderia cepacia* Complex in Nuclease-Free Water and Antiseptics

**DOI:** 10.3390/microorganisms10050943

**Published:** 2022-04-30

**Authors:** Soumana Daddy Gaoh, Ohgew Kweon, Yong-Jin Lee, David Hussong, Bernard Marasa, Youngbeom Ahn

**Affiliations:** 1 Division of Microbiology, National Center for Toxicological Research, U.S. Food and Drug Administration, Jefferson, AR 72079, USA; soumana.daddy-gaoh@fda.hhs.gov (S.D.G.); oh-gew.kweon@fda.hhs.gov (O.K.); 2 Department of Natural Sciences, Albany State University, Albany, GA 31705, USA; yong.lee@asurams.edu; 3 Office of Pharmaceutical Science, Center for Drug Evaluation and Research, U.S. Food and Drug Administration, Beltsville, MD 20993, USA; david.hussong@outlook.com; 4 Office of Pharmaceutical Quality, Center for Drug Evaluation and Research, U.S. Food and Drug Administration, Silver Spring, MD 20993, USA; bernard.marasa@fda.hhs.gov

**Keywords:** *Burkholderia cepacia* complex, propidium monoazide (PMAxx), droplet digital polymerase chain reaction (ddPCR), nuclease-free water, antiseptics

## Abstract

Pharmaceutical products contaminated with *Burkholderia cepacia* complex (BCC) strains constitute a serious health issue for susceptible individuals. New detection methods to distinguish DNA from viable cells are required to ensure pharmaceutical product quality and safety. In this study, we have assessed a droplet digital PCR (ddPCR) with a variant propidium monoazide (PMAxx) for selective detection of live/dead BCC cells in autoclaved nuclease-free water after 365 days, in 0.001% chlorhexidine gluconate (CHX), and in 0.005% benzalkonium chloride (BZK) solutions after 184 days. Using 10 μM PMAxx and 5 min light exposure, a proportion of dead BCC was quantified by ddPCR. The detection limit of culture-based method was 10^4^ CFU/mL, equivalent to 9.7 pg/μL for *B. cenocepacia* J2315, while that of ddPCR was 9.7 fg/μL. The true positive rate from nuclease-free water and CHX using PMAxx-ddPCR assay was 60.0% and 38.3%, respectively, compared to 85.0% and 74.6% without PMAxx (*p* < 0.05), respectively. However, in BZK-treated cells, no difference in the detection rate was observed between the ddPCR assay on samples treated with PMAxx (67.1%) and without PMAxx (63.3%). This study shows that the PMAxx-ddPCR assay provides a better tool for selective detection of live BCC cells in non-sterile pharmaceutical products.

## 1. Introduction

The *Burkholderia cepacia* complex (BCC) is a group of at least 24 closely related species characterized by a high metabolic versatility [1,2,3,4]. BCC bacteria can survive and proliferate under limited nutrient conditions in aqueous environments. They have emerged as one of the most reported contaminants of non-sterile pharmaceutical products posing a major health risk for many susceptible individuals [5]. Several BCC outbreaks have been documented in recent decades, leading to the recall of multiple products [2]. Furthermore, an examination of the U.S. Food and Drug Administration (FDA) Enforcement Reports (2012–2019) demonstrates that BCC is the primary source of non-sterile pharmaceutical product recalls [6,7]. This led the FDA to propose the inclusion of these bacteria in the “Objectionable Microorganisms” category [2,7,8]. Subsequent BCC presence in pharmaceutical products can be attributed to the lack of proper good manufacturing practices (GMP) and the use of compendial methods that lack the sensitivity for BCC detection in pharmaceutical water and other finished products [9].

Traditionally, BCC enumeration has been performed using culture-based procedures that are laborious and time-consuming and exhibit low sensitivity. Molecular techniques, such as PCR, quantitative PCR (qPCR), and droplet digital PCR (ddPCR), have shown great promise in BCC detection due to their pronounced sensitivity and specificity [8,10,11]. Recently, we have shown that ddPCR exhibits more sensitivity in BCC detection in nuclease-free water, chlorhexidine gluconate (CHX), and benzalkonium chloride (BZK) solutions, compared to qPCR (43.5% to 66.8%; 40.0% to 67.0%; and 41.3% to 64.0%, respectively) [9]. The ddPCR is a relatively new technology, of which commercial application started in 2011 [12]. The technology is based on partitioning a sample into thousands of micro-reactions of defined volume prior to amplification. All of the partitions are then amplified to the endpoint and read to determine the fraction of positive partitions [13,14,15]. Based on the number of positive partitions, the amounts of target nucleic acids in the original sample can be calculated directly using the ratio of positives to total partitions by Poisson statistics. The ddPCR assay has been successfully used in various research applications to determine the copy numbers of DNA and RNA in viruses, bacteria, and parasites found in a variety of clinical specimens [16,17]. The ddPCR technology is gaining ground due to its advantages, namely, absolute quantitation of target nucleic acids without the need for a standard curve, better detection of low-copy-number, increased precision at very low template concentrations, less prone to inhibitors, and poor amplification efficiency compared to qPCR. Nonetheless, a major drawback of ddPCR—as all other PCR based assays—is its inability to distinguish between DNA originated from viable cells and DNA from dead cells [18].

Amplification of DNA from nonviable cells will increase the copy number and thus overestimate the bacterial count, which will ultimately provide an unreliable indication of human health risk assessment posed by the presence of pathogens. Propidium monoazide (PMA) has been combined with qPCR to overcome this shortcoming of PCR assays [19,20]. PMA can only penetrate bacterial cells with damaged membranes and covalently bind to double-stranded DNA upon exposure to bright visible light resulting in the amplification of only viable cells [21]. Successful use of the PMA assay has been reported on *Escherichia coli*, *Salmonella enterica*, *Campylobacter coli*, *Brucella suis*, and severe acute respiratory syndrome coronavirus 2 (SARS-CoV-2) in food and water [19,22,23,24,25]. PMAxx, an enhanced version of PMA was introduced in 2015 and showed better discriminative power than PMA between viable and dead cells [26]. Recently, the PMAxx-ddPCR assay has been successfully employed in the detection of trace viable-but-nonculturable (VBNC) *Cronobacter sakazakii* from dairy products, and infant food [27,28], as well as in the quantification of *Lactobacillus rhamnosus* and two *Lactobacillus paracasei* subsp. paracasei strains in piglet faces [29].

To specifically detect viable cells, PMAxx-ddPCR has shown promise. In this study, we developed a PMAxx-ddPCR assay for assessing viable BCC cells in distilled water and antiseptics. BCC strains were added to autoclaved nuclease-free water and cultured for 361 consecutive days (23 January 2020) to 10 μg/mL chlorhexidine gluconate (CHX) and to 50 μg/mL benzalkonium chloride (BZK) for 184 consecutive days (8 September 2020). At first, we optimized conditions for the PMAxx-ddPCR assay, including PMAxx concentration and time of light exposure. After optimization, the performance of the PMAxx-ddPCR assay was validated using nuclease-free water spiked with different ratios of live/dead cells. Finally, the PMAxx-ddPCR assay was applied to study BCC survival in distilled water and antiseptics.

## 2. Materials and Methods

### 2.1. ddPCR Assay

The 20 BCC, 18 non-BCC species and 18 non-*Burkholderia* bacterial strains were used in this research as described previously (Table S1 in the Supplemental Materials) [9,30]. The total genomic DNA was extracted from cultures of the aforementioned test strains using the DNeasy UltraClean Microbial Kit (Qiagen, Valencia, CA, USA), as described previously [30]. The primer sets used for ddPCR in this study are RibB5-F/RibB5-R and RibB67-F/RibB67-R (Table 1), which have proven effective for amplifying the *ribB* genes from all bacterial species tested [30].

The ddPCR assays were carried out on a Bio-Rad QX200™ Droplet Digital™ PCR System, as described previously [9]. Briefly, the reaction mixture was composed of 10 μL of 2 × QX200™ ddPCR™ EvaGreen Supermix (BioRad, Hercules, CA, USA), and 0.3 μM of each primer, and 4 μL of DNA template. Droplets were generated by loading 20 µL of the reaction mixture into the sample well (middle row) of the cartridge and 70 µL of droplet generation oil into the matching oil well (bottom row) of the cartridge, leaving the top row empty. No air bubbles were allowed in the sample or oil. The cartridge was then covered with a DG8™ rubber gasket by hooking the prongs of the cartridge holder through the gasket’s four holes. The whole apparatus was placed into the QX200 droplet generator for droplet generation, which were carefully transferred (around 40 µL droplets into top row of the cartridge) into a 96-well PCR plate (BioRad). The plate was then sealed with a foil at 180 °C for ~5 s, followed by amplification in a T100™ Thermal Cycler (BioRad). The cycling conditions were set as follows: 10 min at 95 °C, followed by 40 cycles of 30 s at 95 °C, 1 min at 60 °C, and then 10 min at 98 °C for droplet stabilization and 4 °C for cooling. After PCR, the 96-well PCR plates were transferred to a droplet reader (BioRad) to detect fluorescent intensities in droplets. QuantaSoft 1.3.2.0 software (BioRad) was used to analyze the DNA concentration from positive reactions using the Poisson distribution. The threshold for a positive signal was set at a fluorescence amplitude of 1500. Only reactions with over 10,000 accepted droplets carried out in quintuplicate were used for analysis [29].

### 2.2. Optimization of PMAxx Treatment

*B. cenocepacia* J2315 cells were grown on 1/10× Trypticase Soy Agar (TSA) at 30 °C for 24 h before being inoculated into autoclaved nuclease-free water (Qiagen) with a final optical density of 0.1 (approximate cell density = 1.5 × 10^8^ colony-forming units (CFU/mL)). Dead cells were obtained from heating 1 mL of suspended cells at 100 °C at 250 rpm for 10 min using the Thermomixer Comfort (Eppendorf, Germany) [22]. The absence of viable cells was confirmed by droplet plating 10 μL aliquots of cells on 1/10× TSA plates and cultured at 30 °C for 30 h before enumeration.

A 20 mM PMAxx (Biotium, Fremont, CA, USA) in autoclaved nuclease-free water (Qiagen) was diluted in nuclease-free water (pH 8.0; Thermo Fisher Scientific Inc., Waltham, MA, USA) to a final 200 µM stock solution. A series of 95, 90, 85, 80, and 75 µL suspensions of live *B. cenocepacia* J2315 (1.5 × 10^8^ CFU/mL) in 1.5-mL microcentrifuge tubes were treated, respectively, with 5, 10, 15, 20, 25 µL of 200 µM PMAxx stock solutions (i.e., final concentrations of 10, 20, 30, 40, and 50 µM PMAxx). The samples were then incubated in the dark with occasional mixing for 5 min to promote PMAxx binding to dead cells. Afterwards, the tubes were exposed to light-emitting diode (LED) light (PMA-Lite; LED Photolysis Device, Biotium E90002) with 465–475 nm emission for 15 min. The genomic DNA was extracted by boiling at 100 °C for 10 min, and the supernatants were used as templates for ddPCR assays. After PMAxx concentration was optimized, light exposure times (i.e., 5, 10, and 15 min) were tested to determine the optimal PMAxx-ddPCR conditions.

To determine the effectiveness of the optimal condition (10 µM PMAxx and 5 min light exposure), a ddPCR assay was performed in the presence of antiseptics. Briefly, two 1.5 mL Eppendorf tubes, each containing 100 µL of *B. cenocepacia* J2315 (1.5 × 10^8^ CFU/mL) cells, were used to extract genomic DNA by the boiling method. DNA concentrations of 25.5 ng/µL and 22.0 ng/µL were determined using a NanoDrop ND-2000 spectrophotometer (Thermo Fisher Scientific Inc.). To determine possible antiseptics interference in ddPCR assay, one-time serial dilution of genomic DNA—equivalent to 2.55 ng/µL and 2.20 ng/µL—was treated, respectively with 1 mg/mL CHX and 2 mg/mL BZK (i.e., final concentrations of 5, 10, 50, and 100 µg/mL CHX, and 10, 50, 100, and 200 µg/mL BZK). DNA samples treated with PMAxx, as well as untreated DNA samples (control) were both subjected to the ddPCR analysis.

### 2.3. Limit of detection (LOD) of PMAxx-ddPCR

Briefly, 100 µL of *B. cenocepacia* J2315 (1.5 × 10^8^ CFU/mL) were boiled for 10 min at 100 °C in a water bath to extract genomic DNA. The genomic DNA was quantified using a NanoDrop ND-2000 spectrophotometer (Thermo Fisher Scientific Inc.). DNA was then serially diluted, ranging from 9.7 ng/µL to 9.7 fg/µL (9.7 ng/µL, 970 pg/µL, 97 pg/µL, 9.7 pg/µL, 970 fg/µL, 97 fg/µL, 9.7 fg/µL) and amplified with the RibB67 primer set by PMAxx-ddPCR to determine the LOD and LOQ of the assay. All assays were performed in triplicate. The LOQ corresponding to the lowest concentration of analyte DNA that provides a reliable and acceptable accuracy, and the LOD corresponding to the lowest concentration of analyte DNA at which the test background can be distinguished.

### 2.4. Assessment of the PMAxx-ddPCR Assay

#### 2.4.1. Using Various Ratios of Live vs. Dead *B. cenocepacia* J2315 Cells

To determine the efficiency of PMAxx in selectively amplifying DNA from viable cells, different compositions of mixed live and dead cells were assessed. Mixtures of live/dead cells were prepared with viable cells constituting 100, 90, 70, 50, 30, and 10% of the total cells of *B. cenocepacia* J2315 (1.5 × 10^8^ CFU/mL). For negative controls, only dead cells (0% viable cells) were used. Each mixture was treated with the PMAxx stock solution (200 µM) to achieve a final concentration of 10 µM PMAxx. The cells were incubated for 5 min in the dark and then exposed to 465–475 nm LED light for 15 min. A total of 100 µL of each mixture was boiled for 10 min in a water bath and used as templates for ddPCR assays.

#### 2.4.2. Using Different Concentrations of CHX and BZK

To assess the effect of the PMAxx-ddPCR assay in presence of antiseptics, 100 µL of live *B. cenocepacia* J2315 (1.5 × 10^8^ CFU/mL) cells were treated with 1 mg/mL CHX, and 2 mg/mL BZK to achieve final concentrations of 5, 10, 50, 100 µg/mL CHX, and 10, 50, 100, 200 µg/mL BZK. The cells were then consecutively treated with PMAxx, and genomic DNA was extracted, as described previously. The concentrations of extracted genomic DNA were assessed using a NanoDrop ND-2000 spectrophotometer (Thermo Fisher Scientific Inc., Waltham, MA, USA) and normalized to 10 ng/μL in nuclease-free water, and 4 µL was used for ddPCR analysis.

### 2.5. Application of the PMAxx ddPCR Assay with 20 BCC Strains in Nuclease-Free Water and Antiseptics

Then, 20 BCC strains with adjusted optical density of 0.1 (1.5 × 10^8^ CFU/mL) were cultured for 361 (23 January 2020) and 184 (8 September 2020) consecutive days in nuclease-free water (Qiagen) and in antiseptics (10 µg/mL for CHX and 50 µg/mL BZK), respectively. Then, 1 mL of each culture was subjected to 10-fold serial dilutions in nuclease-free water to yield 10^4^, 10^3^, 10^2^, 10 CFU/mL. Genomic DNA was extracted using 100 µL sample from the dilutions by boiling at 100 °C for 10 min in a water bath, and 4 µL of DNA were used for the ddPCR assay. Based on the fluorescence amplitude of PMAxx-ddPCR, positive results were recorded (0 = not detected, 1 = detected), and the estimated *sensitivity* was calculated [30].

### 2.6. Statistical Analysis

Fisher’s exact probability tests for 2 × 2 contingency tables using Simple Interactive Statistical Analysis (SISA) web-based software (www.quantitativeskills.com/sisa/statistics/fishrhlp.htm, accessed on 7 March 2022) were used to compare the ddPCR assays with and without PMAxx in nuclease-free distilled water, CHX, and BZK. Significant differences between the detection of BCC cells obtained in CHX and BZK were determined by one-way analysis of variance (ANOVA), Tukey’s post hoc test using the SigmaPlot vers. 13.0 software. The difference was considered statistically significant when *p* ≤ 0.05.

## 3. Results

### 3.1. Evaluating the Primer Sets

Two primers set RibB5 and RibB67 were chosen because of their specificity and sensitivity in BCC detection as reported previously [30]. To evaluate the efficacy of the two primer sets, 20 BCC strains and 36 other bacterial strains (i.e., 18 non-BCC, and 18 non-*Burkholderia*) were initially examined (Supplementary Table S1). The ddPCR assay with either RibB5 or RibB67 successfully amplified all 20 of the BCC strains. All of the other 36 bacterial strains tested negative with the RibB67 primer, except for *Burkholderia* ES0634. However, with the RibB5 primer, ten species of non-BCC (i.e., *Burkholderia* AU12121, *Burkholderia* AU13555, *Burkholderia* AU15822, *Burkholderia* AU16341, *Burkholderia* AU18377, *Burkholderia* AU19944, *Burkholderia* AU29541, *Burkholderia* AU30473, *Burkholderia* AU36262, *Burkholderia* AU37486), and two non-*Burkholderia* strains (i.e., *Pseudomonas aeroginosa* PAO1, *P. aeroginosa* ATCC27853) tested positive. The specificity of RibB5 and RibB67 were 66.7% (24 negative out of 36) and 97.2% (35 negative out of 36), respectively. Therefore, the RibB67 primer set was used in the subsequent PMAxx-ddPCR assay for quantifying viable and non-viable BCC.

### 3.2. Optimization of the PMAxx Concentration and Light Exposure Time

**Figure 1:**
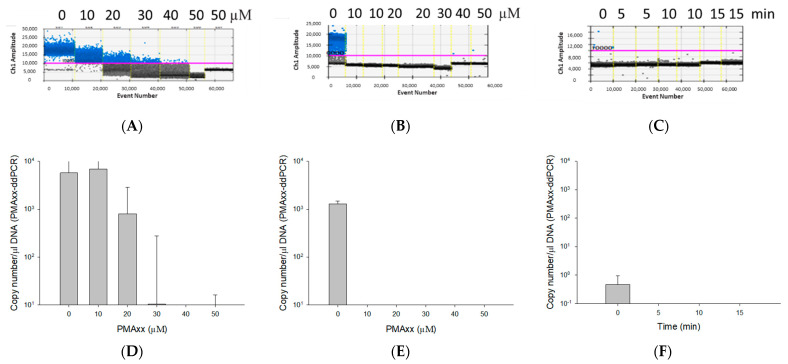
The effect of different concentrations of propidium monoazide (PMAxx) (**A**,**B**,**D**,**E**) and different LED light exposure times (**C**,**F**). The yellow vertical dotted lines separate results of individual reaction wells (**A**–**C**). DNA copy number obtained by ddPCR after exposing live (**D**) and dead (**E**) *B. cenocepacia* J2315 (1.5 × 10^8^ CFU/mL) cells to different PMAxx concentrations (0, 10, 20, 30, 40, and 50 μM PMAxx). DNA copy number obtained by ddPCR after exposing dead *B. cenocepacia* J2315 (1.5 × 10^8^ CFU/mL) to different LED light exposure times (0, 5, 10, and 20 min) (**F**).

The optimal PMAxx concentration was determined by ddPCR after incubating both live and dead cells of *B. cenocepacia* J2315 (1.5 × 10^8^ CFU/mL) for 15 min with five PMAxx concentrations ranging from 10 to 50 µM (Figure 1). The ddPCR results of *B. cenocepacia* J2315 *ribB* gene were obtained from Bio-Rad QX200™ Droplet Reader (Figure 1A–C). The threshold for a positive detection was set at 10,000 relative fluorescence units (rfu). On live cells with 10 µM PMAxx, the assay quantified 4955 copy number/µL DNA, which showed no significant difference compared to 4697 copy number/µL DNA of the control sample (live cells untreated with PMAxx). As we increased the concentration of the PMAxx from 10 to 20 (2041 copy number/µL DNA), 30 (263.5 copy number/µL DNA), 40 (64.9 copy number/µL DNA), and 50 µM (15.3 copy number/µL DNA), we observed a steady decrease in the amplification, with an almost complete inhibition at 50 µM (Figure 1A,D). In contrast, the 10 µM PMAxx affected considerably the dead (i.e., heat-killed) cells (0.44 ± 0.62 copy number/µL DNA) with a complete abolition of the PCR resulting in no molecules being detected (Figure 1B,E). Following this result, 10 µM PMAxx was used on dead *B. cenocepacia* J2315 (1.5 × 10^8^ CFU/mL), and subsequently exposed for 5, 10, 15 min to blue LED light for incubation time optimization. The results showed that within 5 min of LED light exposure, no amplification signal from dead cells were observed, resulting in no molecules being detected (Figure 1C,F). Therefore, a PMAxx concentration of 10 µM and LED light exposure time of 5 min were used in the remaining of the experiments as optimal conditions that promote PMAxx binding to DNA of dead cells to inhibit amplification.

### 3.3. LOD of PMAxx-ddPCR

**Figure 2:**
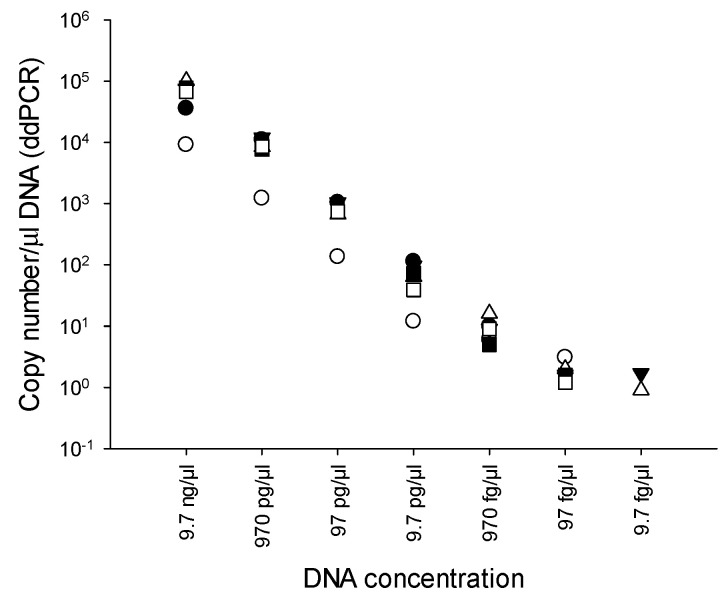
Limit of detection (LOD) of the PMAxx-ddPCR assay. DNA concentrations of the undiluted DNA samples (97 ng/μL) were spectrophotometrically determined at A260.

Ten-fold serial dilutions of genomic DNA of *B. cenocepacia* J2315 (9.7 ng/μL to 9.7 fg/μL) were used to determine the LOD of the PMAxx-ddPCR assay. ddPCR was performed in quintuplicate. The LOD of the PMAxx-ddPCR with RibB67 primers was 9.7 fg/μL, equivalent to 0.9 to 1.7 copies number/μL DNA (Figure 2). Meanwhile, the limit of quantification (LOQ) was determined as was the LOD. The LOQ of the PMAxx-ddPCR with the RibB67 primers was 97 fg/μL. On the basis of this LOQ observation, we used the aforementioned 97 fg/μL DNA value in the remaining experiments.

### 3.4. Assessment of the PMAxx-ddPCR Assay

#### 3.4.1. Assessing Live/Dead Cells in Nuclease-Free Water

**Figure 3:**
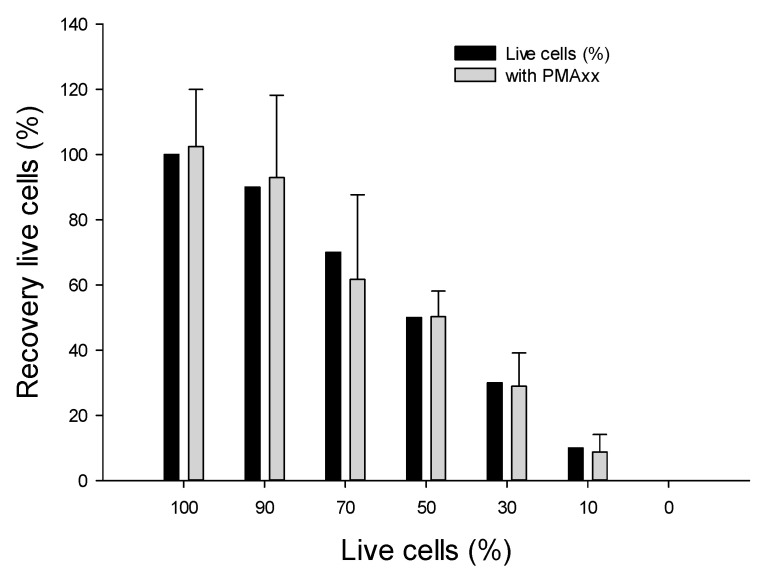
Effectiveness of PMAxx in selectively amplifying DNA from viable cells, mixtures of viable and dead cells were evaluated. Percentage of viable cells corresponded to 100, 90, 70, 50, 30, 10, 0%, after 10 µM PMAxx treatment.

We assessed the effectiveness of the PMAxx-ddPCR assay using the optimal condition of 10 µM PMAxx and 5 min of LED light exposure, as described earlier. A mixture of viable and dead cells corresponding to 100, 90, 70, 50, 30, 10, and 0% (live/dead ratios) were subjected to the PMAxx-ddPCR assay (Figure 3). The 100% viable cells were 102.4 ± 17.6% (average 5368 copy number/µL DNA). Based on these results, the ratio was calculated relatively in comparison with 100% viable cells. The calculated recovery rates of the mixtures with 90% and 70% live/dead ratios in the PMAxx-ddPCR assay were 92.8% (4986 copy number/µL DNA), and 61.7% (3311.4 copy number/µL DNA), respectively. The recovery rates of mixtures with 50, 30, and 10% live/dead ratios in the PMAxx-ddPCR assay were found to be 50.3% (2698 copy number/µL DNA), 28.9% (1552.8 copy number/µL DNA), and 8.7% (467 copy number/µL DNA), respectively. The mean recovery percentage of live cells decreased gradually with increasing proportion of dead cells. This indicates that the DNA from dead cells was effectively inactivated by PMAxx treatment. Indeed, control samples consisting of PMAxx™-treated dead cells (0% live cells) yielded no detectable signal with ddPCR. Therefore, the PMAxx-ddPCR assay accurately detected viable cells from mixtures of viable and non-viable cells. False positive amplifications remained very limited.

#### 3.4.2. PMAxx-ddPCR Assay in CHX and BZK

**Figure 4:**
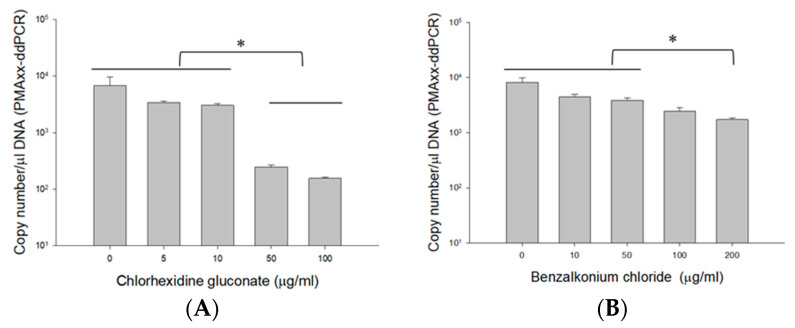
DNA copy number obtained by ddPCR in live *B. cenocepacia* J2315 (1.5 × 10^8^ CFU/mL) cells efficiency of suspensions spiked with 5, 10, 50, 100 µg/mL CHX (**A**), and 10, 50, 100, 200 µg/mL BZK (**B**). Error bars in diagrams represent the mean value ± standard deviations obtained in triplicates. The * symbol over each diagram indicates a statistically significant difference, *p* < 0.05.

To demonstrate the applicability of the PMAxx-ddPCR assay on antiseptics, *B. cenocepacia* J2315 (1.5 × 10^8^ CFU/mL) cells were quantified in CHX and BZK (Figure 4). The mean PMAxx-ddPCR assay quantified without CHX (6766.7 copy number/μL DNA) was like those observed in 5 (3390 copy number/μL DNA) and 10 µg/mL CHX (3056.7 copy number/μL DNA) (Figure 4A). ANOVA indicated that the quantification of BCC obtained without CHX did not differ (*p* > 0.057) from the quantification observed in 0, 5, and 10 µg/mL CHX. However, the copy numbers obtained from 50 (248.67 ± 20.31 copy number/μL DNA) and 100 µg/mL CHX (156.33 ± 6.43 copy number/μL DNA) were 10-fold lower than those from 0 to 10 µg/mL CHX.

Similar results were also obtained with BZK samples. The mean PMAxx-ddPCR assay quantified without BZK (8163.3 copy number/μL DNA) was similar to those obtained in 10 (4436.7 copy number/μL DNA) and 50 µg/mL BZK (3833.3 copy number/μL DNA) (Figure 4B). However, the copy numbers obtained from 100 (2470 copy number/μL DNA) and 200 µg/mL BZK (1724.7 copy number/μL DNA) were two-fold lower than those quantified in 0 to 50 µg/mL BZK. ANOVA indicated that the quantification of BCC obtained without BZK was significantly different (*p* < 0.019) from the quantification observed in 100 and 200 µg/mL BZK. These results indicate a potential interference at higher concentrations of CHX (≥50 µg/mL) and BZK (≥200 µg/mL). This interference could potentially limit the accuracy of the viability assay. On the basis of these results, we confirmed that using concentrations of 10 µg/mL CHX and 50 µg/mL BZK would be suitable for the PMAxx-ddPCR assay in antiseptics.

### 3.5. Application of PMAxx-ddPCR Assay in Nuclease-Free Water and Antiseptics

We assessed the sensitivity (true-positive rate) of the PMAxx-ddPCR assay using 20 BCC strains cultured for 365 days in nuclease-free water and 184 days in 10 µg/mL for CHX and 50 µg/mL BZK. A total of 240 samples (20 strains × 4 serial dilutions × 3 replicates) of PMAxx treated (live cells) and untreated (total cells) corresponding to 10, 10^2^, 10^3^, and 10^4^ CFU/mL were analyzed by ddPCR. Table 2 shows a sensitivity comparison of ddPCR assays on both untreated and PMAxx-treated samples.

The PMAxx-ddPCR assay could detect all serially diluted inoculations (≥10 CFU/mL of BCC) from autoclaved nuclease-free water samples. Among 240 tests in nuclease-free water without PMAxx treatment, 204 were true positives, while 36 were false negatives, which yielded a sensitivity of 85.0% (TP/(TP + FN) × 100 = 204/(204 + 36) × 100). In comparison, the sensitivity of the PMAxx treated sample was 60.0%, with 144 true positives and 96 false negatives (TP/(TP + FN) × 100 = 144/(144 + 96) × 100). Although the ddPCR assay without PMAxx showed a slightly higher sensitivity compared to the PMAxx-ddPCR assay at all serial dilution samples, no significant difference (*p* > 0.05) was observed (Supplementary Table S2).

In 10 µg/mL CHX samples incubated at 23 °C for 184 days, the true positive rate without and with PMAxx were 74.6% (179 out of 240) and 38.3% (92 out of 240) (*p* = 0.0001), respectively (Table 2). Interestingly, in lower dilution samples (at 10, 10^2^, and 10^3^ CFU/mL), the sensitivity of PMAxx-ddPCR assay (26.7%, 18.3%, and 38.3%) was significantly less (*p* < 0.05) than those of without PMAxx (58.3%, 68.3%, and 81.7%). However, in 10^4^ CFU/mL dilution samples, the sensitivity of PMAxx-ddPCR assay (70.0%) was not significantly different (*p* = 0.4112) from that of ddPCR without PMAxx (90%) (Supplementary Table S3).

The ddPCR assay with 50 µg/mL BZK samples incubated at 23 °C for 184 days was determined without PMAxx and showed 63.33% (152 positives out of 240) true positive rate, which were similar to those obtained with PMAxx (67.08%; 161 positives out of 240) (*p* = 0.7166) (Table 2). In all of those dilutions, there is a significant difference between PMAxx-treated and untreated cells. The sensitivity of PMAxx-ddPCR was over 40% (24 out of 60) in 10 and 10^2^ CFU/mL samples, while 10^3^ and 10^4^ CFU/mL samples were above 83.3% (50 out of 60) (Supplementary Table S4).

## 4. Discussion

BCC bacteria are a major health concern due to their ability of surviving in harsh environmental conditions and evading detection. They have established themselves as a prominent objectionable organism associated with 45.3% of non-sterile drug recalls in recent years [6]. Even though there are various nucleic acid-based BCC detection methods, their inability to distinguish between viable and non-viable BCC remains challenging. Here, we have developed and evaluated a PMAxx-ddPCR assay using the RibB67 primer set for selective detection of live BCC from nuclease-free water and antiseptics.

Genetically, the BCC is a very diverse group of bacteria, and its molecular detection rely mostly on the *recA* gene [10]. In fact, BCC microorganisms have a *recA* gene sequence comparability of 94 to 95% between species and 98 to 99% between sequences of the same species, making it a viable candidate for species identification [2,10]. The *ribB*-based BCC-specific loop-mediated isothermal amplification assay (LAMP) primers (RibB5, RibB16, and RibB67) were previously developed [30]. Both BLAST analysis and full-scale lab trials were used to confirm their specificity and sensitivity. RibB5, RibB16, and RibB67 had 100%, 63.9%, and 86.1% specificity, respectively, according to the LAMP findings. As a result, we chose RibB5 and RibB67 for ddPCR. RibB67 ddPCR primer set had a specificity of 97.2% in this study, while RibB5 ddPCR primer set had a specificity of 66.7%. The complexity of LAMP, which employs four primer sequences plus two loop primers, explains the difference in specificity between LAMP and ddPCR. LAMP’s large number of primers may raise the chance of primer dimerization, lowering specificity [31]. The RibB5 ddPCR primer set only contains two separate locations on the target nucleotide sequence, increasing the possibility of cross homology-induced false-positive amplification. Meanwhile, the elimination of degenerated primers may account for the excellent specificity of RibB67 ddPCR primers. The latter result in well-known significant, but non-specific amplification [32]. The multiple primers—or the degenerated primer set—are necessary to cover highly divergent genes within the same functional groups. In contrast to the RibB5 ddPCR primer, the RibB67 ddPCR primer pair enabled specific detection of BCC from nuclease-free water and antiseptics.

Our primary focus on the current study was to discriminate live BCC cells from dead BCC cells found in nuclease-free water and antiseptics. We have combined ddPCR with PMAxx to overcome a major drawback of the conventional assay in its inability to differentiate between DNA from viable and non-viable cells. Two fundamental factors, namely suitable PMA concentration and proper light exposure time are critical for discrimination between viable (live) and non-viable (dead) cells, as previously reported [21,33,34]. Thus, different PMAxx concentrations and various time exposures were investigated to determine the optimal condition for the ddPCR assay. In this study, 10 µM PMAxx treatment on BCC cultures, followed by a 5 min LED light exposure was sufficient to discriminate between viable and non-viable BCC cells. Similarly, an optimal condition of 8–10 µM PMAxx and 10 min light exposure was effective in reducing the signal originating from heat inactivated *C. sakazakii* [28]. A previous study reported that 25 µM PMAxx and a 15 min light exposure was needed to distinguish between viable and non-viable *Salmonella* cells [20]. In contrast, a higher PMA concentration (100 µM) with 5–15 min light exposure times was required for measuring viable lactic acid bacteria [29]. Similarly, 50 and 100 µM PMA with 15 min photoactivation were required to discriminate intact viruses from heat inactivated human noroviruses (HuNoV GI and GII) [26]. However, our findings showed that a higher PMAxx concentration significantly reduced the amplification of DNA from live cells. Indeed, the amplification from live cells with 10 µM PMAxx treatment (4955 copy number/μL DNA) decreased over 50% compared to that with 20 µM PMAxx treatment (2041 copy number/μL DNA). The proportion of viable cells decreased as PMAxx concentration increased up to 50 μM. These results suggest a potential cytotoxic effect of PMAxx in viable BCC cells as described for bacteria such as *Listeria monocytogenes* and *Legionella pneumophila* [33].

We also investigated the potential interference of CHX and BZK in the PMAxx-ddPCR assay since a different matrix would considerably affect the efficiency of live/dead assays [29,33]. In this study, 5–100 µg/mL CHX and 10–200 µg/mL BZK solutions did not affect the ddPCR assay (data not shown). However, higher concentrations of CHX (≥50 µg/mL) and BZK (≥200 µg/mL) affected the viability of live cells based on the PMAxx-ddPCR assay. This may be due to susceptible concentrations of 50–500 µg/mL (CHX) and 10–200 µg/mL (BZK) at initial inoculation and an incubation time of 40 days after inoculating in water [35]. Since CHX and BZK impact membrane permeability, causing leakage of cytoplasmic materials at low concentrations due to damage to the outer membrane, CHX and BZK have a significant effect on cell viability [36]. As reported by Cangelosi et al. [37], most cell membranes permeable to PMA are probably those of non-viable cells, but cell membranes of some non-viable cells may still be intact. Our results suggested that applying 10 µM PMAxx and a 5 min LED light exposure was sufficient to facilitate screening of viable BCC cells, especially in nuclease-free water and in 10 µg/mL CHX, as well as 50 µg/mL BZK solutions.

The PMAxx-ddPCR assay with the RibB67 primer showed an LOD of 9.7 fg/mL which was a 100-fold more sensitive in BCC detection compared to our recent finding using the RibB5 primer with LAMP assay [30]. Furthermore, the PMAxx-ddPCR assay showed a tenfold greater sensitivity than qPCR (103 fg/mL). In comparison, these results were consistent with previous BCC detection studies, in which ddPCR showed a tenfold greater sensitivity than qPCR for detecting *B. cepacia* PC783 and *B. cenocepacia* J2315 strains in nuclease-free water and antiseptics [9]. The PMAxx-ddPCR assay in nuclease-free water and antiseptics was able to quantify as few as 10 CFU/mL of BCC, which corresponds to an LOD of 9.7 fg/mL. Our results are consistent with earlier findings where we demonstrated 23 CFU/mL for detecting VBNC *C. sakazakii* in pure culture [27]. Wang et al. reported ddPCR showed detection limits as low as 250 CFU/mL for *Salmonella* Typhimurium in milk [31]. Previously, Cremonesi et al. [38] also achieved a higher sensitivity of 100 CFU/g for the *Listeria* spp. assay in soft cheese. Our results indicate that the established PMAxx-ddPCR method provides enough sensitivity in detecting viable BCC cells from nuclease-free water and antiseptics and is more sensitive than qPCR.

ddPCR has been proven to be a good detection method for BCC with a sensitivity of 64–67% in nuclease-free water, CHX, and BZK [9]. In this study, ddPCR of BCC cells cultured in distilled water and antiseptics consistently showed a sensitivity range of 63–85%. When PMAxx was combined with ddPCR, the sensitivity was lower in nuclease-free water (60%) and CHX samples (38%), but not in BZK samples (67%). This indicates that PMAxx can be used as a nucleic acid dye to rule out the effects of amplifying dead bacteria [27,28]. Indeed, the recovery percentage of live cells with PMAxx decreased gradually as the proportion of dead cells increased. Although, ddPCR is more labor-intensive, time-consuming, and costly, it can be used for detecting with a high degree of sensitivity, low numbers of cells. The PMAxx-ddPCR assay developed in the present study represents a new strategy to quantify live BCC cells directly in non-sterilized pharmaceutical products.

## 5. Conclusions

We report a rapid, sensitive, and specific PMAxx-ddPCR assay for the detection of viable BCC cells in nuclease-free water and antiseptics. To our knowledge, this represents an early attempt of viability assay using ddPCR in detecting live BCC cells. The PMAxx-ddPCR assay was optimized, validated, and applied successfully in studying 20 BCC strains cultured for 365 days in nuclease-free water and 184 days in 10 µg/mL CHX and 50 µg/mL BZK. Our viability assay using PMAxx-ddPCR may provide a practical insight in determining the presence of BCC cells in pharmaceutical products and offer a tool for a health risk-based assessment in clinical settings.

## Figures and Tables

**Table 1:** Primer sequences for ddPCR.

Amplicons Name		Primer Sequence (5—3)	Amplicon Size (bp)
RibB67	Forward Reverse	GCGATACGAAGGAACACCTG CGTAGCCGGACATGCTG	189
RibB5	Forward Reverse	GGCCGGATGGTGATCCT GTCATCAGCGGCAGGTG	176

**Table 2:** Estimated sensitivity analysis of the PMAxx- ddPCR assay from BCC cultured from nuclease-free distilled water, CHX, and BZK.

Tested Inoculum (CFU/mL)	Nuclease-Free Distilled Water		CHX		BZK	
	Without PMAxx	With PMAxx	Without PMAxx	With PMAxx	Without PMAxx	With PMAxx
10^4^	50/60 ^a^	38/60	54/60	42/60	48/60	50/60
10^3^	55/60	34/60	49/60	23/60	47/60	54/60
10^2^	49/60	37/60	41/60	11/60	28/60	33/60
10	50/60	35/60	35/60	16/60	29/60	24/60
	204/240 (85.0%) ^b^	144/240 (60.0%)	179/240 (74.6%)	92/240 (38.3%)	152/240 (63.3%)	161/240 (67.1%)
	*p* = 0.0163 ^c^		*p* = 0.0001		*p* = 0.7166	

^a^ Number of positive/number of tests. ^b^ Estimated sensitivity (%) = number of true positive events/(number of true positive events + number of false negative events) × 100. ^c^ There is a statistically significant difference between untreated and PMAxx-treated samples (*p* < 0.05).

## Data Availability

The data presented in this study are available on request from the corresponding author.

## Supplementary Materials

The following are available online at https://www.mdpi.com/article/10.3390/microorganisms10050943/s1, Table S1: Specificity analysis using RibB5 and RibB67 primer on 20 BCC, 18 Non-BCC, and 18 non-*Burkholderia* bacterial strains. Table S2: Comparison of positive results for *B. cepacia* complex without PMAxx and with PMAxx in nuclease-free water. Table S3: Comparison of positive results for *B. cepacia* complex without PMAxx and with PMAxx in CHX. Table S4: Comparison of positive results for *B. cepacia* complex without PMAxx and with PMAxx in BZK.
