# Supplementary material for: A Propidium Monoazide (PMAxx)-Droplet Digital PCR (ddPCR) for the Detection of Viable Burkholderia cepacia Complex in Nuclease-Free Water and Antiseptics
Source: Microorganisms. 2022 Apr 30;10(5):943. doi: 10.3390/microorganisms10050943 (PMC9147393; doi:10.3390/microorganisms10050943)
Supplement: Supplementary file 1 [file microorganisms-10-00943-s001.zip › microorganisms-1692048-supplementary.pdf]

SUPPLEMENTARY MATERIAL

Table S1. Specificity analysis using RibB5 and RibB67 primer on 20 BCC, 18 Non-BCC, and 18 non-*Burkholderia* bacterial strains.

Bacteria	No	Strain name	Source	DNA concentration (ng/μL)	Primer	
					RibB67	RibB5
BCC	1	*B. cepacia* PC783	Onion	917.3	+[a]	+
	2	*B. cepacia* AU24442	[b]CF sputum	856.8	+	+
	3	*B. stabilis* AU23340	CF sputum	980	+	+
	4	*B. pyrrocinnia* AU11057	CF sputum	173	+	+
	5	*B. ambifaria* HI2468	Pea, rhizosphere	372	+	+
	6	*B. anthina* HI2738	Soil rhizosphere	454.7	+	+
	7	*B. metallica* AU0553	CF sputum	66.2	+	+
	8	*B. metallica* AU16697	CF sputum	121	+	+
	9	*B. contaminans* HI3429	Sheep with mastitis, milk	246.6	+	+
	10	*B. contaminans* AU24637	CF lung	973.8	+	+
	11	*B. diffusa* AU1075	CF sputum	1481	+	+
	12	*B. arboris* ES0263a	Soil	726	+	+
	13	*B. arboris* AU22095	CF sputum	1220	+	+
	14	*B. lata* HI4002	Forest soil	1095.8	+	+
	15	*B. cenocepacia* AU1054	CF blood	469.3	+	+
	16	*B. cenocepacia* AU0222	CF patient	655.7	+	+
	17	*B. cenocepacia* AU19236	CF sputu	687.4	+	+
	18	*B. cenocepacia* HI2976	Environment, sink	344.1	+	+
	19	*B. cenocepacia* HI2485	CDC sample	657	+	+
	20	*B. cenocepacia* J2315	CF Sputum	473.8	+	+
Non-BCC	21	*B. glumae* AU6208	CF lung nodule	611.8	-	-
	22	*B. plantarii* AU9801	CF Sputum	834.4	-	-
	23	*Caballeronia zhejiangensis* AU10475	CF Blood	1382.8	-	-
	24	*Caballeronia zhejiangensis* AU12096	CF Blood	1657.9	-	-
	25	*B. concitans* AU12121	CF Sputum	635.3	-	+
	26	*B. lumae* AU12450	Non-CF Lung	523.2	-	-
	27	*B. thailandensis* AU13555	Non CF infant Blood (wolfs parkinson white)	541	-	+
	28	*B. tropica* AU15822	CF Throat	435.9	-	+
	29	*B. gladioli* AU16341	CF Sputum	99.1	-	+
	30	*B. fungorum* AU18377	Non CF Jaw Aspirate	782.2	-	+
	31	*B. tropica* AU19944	CFSputum-Infant	859.6	-	+
	32	*B. gladioli* AU26454	CF Sputum	688.7	-	-
	33	*B. gladioli* AU29541	CF Sputum	599	-	+
	34	*B. gladioli* AU30473	CF Sputum	360.3	-	+
	35	*B. fungorum* AU35949	CF Endotracheal	830.4	-	-
	36	*B. thailandensis* AU36262	CF Sputum	527.8	-	+
	37	*B. plantarii* AU37486	CF Sputum	1402.6	-	+
	38	*B. oklahomensis* ES0634	Environmental	362.3	+	-
Non-*Burkholderia*	39	*Enterococcus faecalis* ATCC29212		646	-	-
	40	*Enterococcus durans* ATCC6056		245	-	-
	41	*Proteus mirabilis* ATCC7002		1734.6	-	-
	42	*Enterococcus faecium* ATCC35667		244.5	-	-
	43	*Bacillus subtilis* ATCC6051		1641.6	-	-
	44	*Citrobacter frendii* ATCC8090		2070.6	-	-
	45	*Pseudomonas aeroginosa* PAO1		1782	-	+
	46	*Enterococcus faecium* ATCC49624		3483.5	-	-
	47	*Yersinia enterocolitica subsp.entrocolitica* ATCC27729		1911	-	-
	48	*Shigella sonnei* ATCC9290		1878	-	-
	49	*Lactobacillus salivarius subsp. Salivarius* ATCC11741		1533	-	-
	50	*Enterobacter aerogenes* ATCC13048		1214.7	-	-
	51	*Klebsiella pneumoniae* ATCC13883		1590.5	-	-
	52	*Pseudomonas aeroginosa* ATCC27853		1805.7	-	+
	53	*Candida albicans* (Robin) Berkhout ATCC10231		53	-	-
	54	*Salmonella enterica*		1984.9	-	-
	55	*Paenibacillus lautus*		1479.9	-	-
	56	*Brevibacillus laterosporus*		1507.5	-	-

[a]+: positive reaction, -: negative reaction

[b]CF: cystic fibrosis

Table S2. Comparison of positive results for *B. cepacia* complex without PMAxx and with PMAxx in nuclease-free water.

		Inoculum (CFU/ml)							
		10		10^2		10^3		10^4	
		without PMAxx	with PMAxx	without PMAxx	with PMAxx	without PMAxx	with PMAxx	without PMAxx	with PMAxx
1	*Burkholderia cepacia* PC783	2/3[a]	2/3	2/3	2/3	2/3	0/3	3/3	3/3
2	*Burkholderia cepacia* AU24442	2/3	3/3	2/3	2/3	3/3	3/3	1/3	3/3
3	*Burkholderia stabilis* AU23340	2/3	3/3	2/3	2/3	2/3	1/3	1/3	3/3
4	*Burkholderia pyrrocinia* AU11057	3/3	0/3	3/3	0/3	3/3	1/3	2/3	2/3
5	*Burkholderia ambifaria* HI2468	2/3	3/3	2/3	3/3	3/3	2/3	2/3	2/3
6	*Burkholderia anthina* HI2738	3/3	3/3	3/3	3/3	0/3	2/3	2/3	1/3
7	*Burkholderia metallica* AU0553	3/3	3/3	2/3	3/3	3/3	2/3	3/3	1/3
8	*Burkholderia metallica* AU16697	3/3	3/3	3/3	2/3	3/3	2/3	3/3	2/3
9	*Burkholderia contaminans* HI3429	3/3	3/3	3/3	3/3	3/3	2/3	3/3	2/3
10	*Burkholderia contaminans* AU24637	2/3	0/3	2/3	0/3	3/3	3/3	3/3	2/3
11	*Burkholderia diffusa* AU1075	3/3	3/3	3/3	3/3	3/3	3/3	3/3	2/3
12	*Burkholderia arboris* ES0263[a]	2/3	3/3	3/3	3/3	3/3	3/3	1/3	2/3
13	*Burkholderia arboris* AU22095	3/3	2/3	3/3	3/3	3/3	3/3	2/3	3/3
14	*Burkholderia lata* HI4002	2/3	1/3	2/3	3/3	3/3	3/3	3/3	2/3
15	*Burkholderia cenocepacia* AU1054	3/3	3/3	3/3	3/3	3/3	2/3	3/3	3/3
16	*Burkholderia cenocepacia* AU0222	1/3	0/3	3/3	1/3	3/3	0/3	3/3	0/3
17	*Burkholderia cenocepacia* AU19236	3/3	0/3	2/3	0/3	3/3	0/3	3/3	2/3
18	*Burkholderia cenocepacia* HI2976	3/3	0/3	2/3	0/3	3/3	0/3	3/3	1/3
19	*Burkholderia cenocepacia* HI2485	2/3	0/3	1/3	1/3	3/3	0/3	3/3	0/3
20	*Burkholderia cenocepacia* J2315	3/3	0/3	3/3	0/3	3/3	2/3	3/3	2/3
**Total**		**50/60 (83.3%)** $p = 0.2556$	**35/60 (58.3%)**	**49/60 (81.7%)** $p = 0.3956$	**37/60 (61.7%)**	**55/60 (91.7%)** $p = 0.0942$	**34/60 (56.7%)**	**50/60 (83.3%)** $p = 0.3991$	**38/60 (63.3%)**

[a]Number of positive/number of tests

Table S3. Comparison of positive results for *B. cepacia* complex without PMAxx and with PMAxx in CHX.

		Inoculum (CFU/ml)							
		10		10^2		10^3		10^4	
		without PMAxx	with PMAxx	without PMAxx	with PMAxx	without PMAxx	with PMAxx	without PMAxx	with PMAxx
1	*Burkholderia cepacia* PC783	1/3	1/3	0/3	0/3	2/3	0/3	1/3	0/3
2	*Burkholderia cepacia* AU24442	0/3	0/3	2/3	0/3	2/3	0/3	3/3	0/3
3	*Burkholderia stabilis* AU23340	2/3	1/3	2/3	0/3	3/3	0/3	3/3	0/3
4	*Burkholderia pyrrocinia* AU11057	2/3	1/3	2/3	0/3	3/3	3/3	3/3	2/3
5	*Burkholderia ambifaria* HI2468	1/3	2/3	1/3	1/3	2/3	0/3	3/3	2/3
6	*Burkholderia anthina* HI2738	3/3	2/3	3/3	2/3	3/3	2/3	3/3	3/3
7	*Burkholderia metallica* AU0553	2/3	3/3	2/3	2/3	1/3	2/3	3/3	3/3
8	*Burkholderia metallica* AU16697	2/3	0/3	3/3	1/3	3/3	2/3	3/3	3/3
9	*Burkholderia contaminans* HI3429	1/3	0/3	2/3	1/3	3/3	2/3	3/3	2/3
10	*Burkholderia contaminans* AU24637	3/3	1/3	3/3	0/3	1/3	1/3	3/3	3/3
11	*Burkholderia diffusa* AU1075	0/3	1/3	2/3	2/3	3/3	0/3	2/3	0/3
12	*Burkholderia arboris* ES0263[a]	1/3	0/3	1/3	0/3	2/3	1/3	2/3	3/3
13	*Burkholderia arboris* AU22095	1/3	1/3	2/3	0/3	3/3	0/3	3/3	2/3
14	*Burkholderia lata* HI4002	1/3	0/3	1/3	0/3	2/3	1/3	3/3	1/3
15	*Burkholderia cenocepacia* AU1054	3/3	2/3	2/3	1/3	3/3	1/3	3/3	3/3
16	*Burkholderia cenocepacia* AU0222	1/3	0/3	3/3	1/3	3/3	2/3	3/3	3/3
17	*Burkholderia cenocepacia* AU19236	2/3	1/3	2/3	0/3	3/3	0/3	3/3	3/3
18	*Burkholderia cenocepacia* HI2976	3/3	0/3	3/3	0/3	3/3	3/3	3/3	3/3
19	*Burkholderia cenocepacia* HI2485	3/3	0/3	3/3	0/3	1/3	2/3	2/3	3/3
20	*Burkholderia cenocepacia* J2315	3/3	0/3	2/3	0/3	3/3	1/3	2/3	3/3
**Total**		**35/60*** **(58.3%)** $p = 0.0292$	**16/60** **(26.7%)**	**41/60*** **(68.3%)** $p = 0.0004$	**11/60** **(18.3%)**	**49/60*** **(81.7%)** $p = 0.0164$	**23/60** **(38.3%)**	**54/60** **(90.%)** $p = 0.4112$	**42/60** **(70.0%)**

[a]Number of positive/number of tests

*There is a statistically significant difference between the without PMAxx and with PMAxx ($p < 0.05$).

Table S4. Comparison of positive results for *B. cepacia* complex without PMAxx and with PMAxx in BZK.

		Inoculum (CFU/ml)							
		10		10^2		10^3		10^4	
		without PMAxx	with PMAxx	without PMAxx	with PMAxx	without PMAxx	with PMAxx	without PMAxx	with PMAxx
1	*Burkholderia cepacia* PC783	2/3	3/3	2/3	2/3	2/3	3/3	1/3	3/3
2	*Burkholderia cepacia* AU24442	3/3	3/3	3/3	3/3	2/3	2/3	3/3	2/3
3	*Burkholderia stabilis* AU23340	2/3	3/3	1/3	2/3	2/3	2/3	2/3	3/3
4	*Burkholderia pyrrocinia* AU11057	2/3	0/3	1/3	2/3	3/3	3/3	3/3	2/3
5	*Burkholderia ambifaria* HI2468	1/3	1/3	1/3	3/3	3/3	3/3	3/3	3/3
6	*Burkholderia anthina* HI2738	3/3	3/3	0/3	3/3	3/3	2/3	3/3	2/3
7	*Burkholderia metallica* AU0553	3/3	3/3	3/3	3/3	3/3	3/3	3/3	3/3
8	*Burkholderia metallica* AU16697	3/3	2/3	3/3	3/3	3/3	3/3	3/3	3/3
9	*Burkholderia contaminans* HI3429	0/3	0/3	2/3	2/3	3/3	3/3	3/3	1/3
10	*Burkholderia contaminans* AU24637	0/3	2/3	1/3	3/3	3/3	3/3	3/3	2/3
11	*Burkholderia diffusa* AU1075	1/3	0/3	2/3	0/3	3/3	3/3	3/3	3/3
12	*Burkholderia arboris* ES0263[a]	2/3	0/3	1/3	0/3	3/3	3/3	3/3	2/3
13	*Burkholderia arboris* AU22095	1/3	0/3	1/3	0/3	2/3	2/3	1/3	3/3
14	*Burkholderia lata* HI4002	0/3	0/3	0/3	2/3	1/3	3/3	2/3	2/3
15	*Burkholderia cenocepacia* AU1054	0/3	3/3	0/3	3/3	3/3	3/3	2/3	3/3
16	*Burkholderia cenocepacia* AU0222	2/3	1/3	1/3	1/3	1/3	3/3	3/3	3/3
17	*Burkholderia cenocepacia* AU19236	1/3	0/3	1/3	0/3	2/3	2/3	3/3	2/3
18	*Burkholderia cenocepacia* HI2976	0/3	0/3	1/3	1/3	1/3	3/3	0/3	3/3
19	*Burkholderia cenocepacia* HI2485	3/3	0/3	2/3	0/3	2/3	3/3	2/3	3/3
20	*Burkholderia cenocepacia* J2315	0/3	0/3	2/3	0/3	2/3	2/3	2/3	2/3
**Total**		**29/60 (48.3%)** $p = 0.6222$	**24/60 (40.0%)**	**28/60 (46.7%)** $p = 0.6392$	**33/60 (55.0%)**	**47/60 (78.3%)** $p = 0.6855$	**54/60 (90.0%)**	**48/60 (80.0%)** $p = 0.8925$	**50/60 (83.3%)**

[a]Number of positive/number of tests
